# Low digit ratio (2D:4D) in male suicide victims

**DOI:** 10.1007/s00702-016-1608-4

**Published:** 2016-08-26

**Authors:** Bernd Lenz, Daniela Thiem, Polyxeni Bouna-Pyrrou, Christiane Mühle, Christina Stoessel, Peter Betz, Johannes Kornhuber

**Affiliations:** 1 Department of Psychiatry and Psychotherapy, Friedrich-Alexander University Erlangen-Nürnberg (FAU), Schwabachanlage 6, 91054 Erlangen, Germany; 2 Institute for Legal Medicine, Friedrich-Alexander University Erlangen-Nürnberg (FAU), Erlangen, Germany

**Keywords:** Prenatal androgen exposure, Suicide, Second-to-fourth-finger length ratio, 2D:4D

## Abstract

Although women attempt suicides equally or more often than men do, men are more likely to die of suicide than women (sex paradox of suicidal behavior). Furthermore, the male traits of aggression and impulsivity predict suicide completion. Here, we studied the second-to-fourth-finger length ratio (2D:4D), a proxy for prenatal androgen exposure, in 46 suicide corpses and 25 non-suicide corpses. We report significantly lower 2D:4D in male suicide corpses than non-suicide corpses (*p* = .030, partial *η* ^2^ = .147). There was no significant association between 2D:4D and the suicide method. Our findings indicate increased risk of suicide following higher prenatal androgen exposure in males. The results may improve future efforts to predict and prevent suicides.

## Introduction

The World Health Organization (WHO) estimates that 804,000 individuals worldwide committed suicide in 2012, which corresponds to an age-standardized suicide rate of approximately 11 per 100,000 per year. Reducing the suicide rate is a global goal (WHO 2014). Although many demographic and clinical factors, such as psychiatric illness, male sex, age, and prior suicide ideation and attempts, are established identifiers of people at risk for suicide, these factors are insufficient at predicting which individuals will effectively complete suicide (Goldstein et al. 1991; Nock et al. 2008). Additional knowledge about the mechanisms underlying the occurrence of suicidal ideation and the transition to a completed suicide is necessary to develop and optimize predictive and preventive strategies.

Males are more likely to die of suicide than females. The global age-standardized male-to-female ratio is 1.9 and can be as high as 3.5 for high-income countries (WHO 2014). By contrast, females attempt suicides equally or more often than males do, and the male-to-female ratio varies between .5 and 1.0 depending on region (WHO 2014). Different socioeconomic (e.g., family income and family structure), psychological (e.g., gender roles and behavioral traits), and biological [e.g., genetics and epigenetics (Labonte and Turecki 2010)] factors account for this sex paradox of suicidal behavior. In accordance with the hypothesis that the male traits of aggression and impulsivity facilitate suicidal behavior, these behaviors were reported to predict suicidal attempts and suicide completion (Klonsky and May 2010; Gvion and Apter 2011; Wang et al. 2014; Chachamovich et al. 2015). Prenatal exposure to androgens is a determinant of aggressive behavior in later life (Bailey and Hurd 2005; Joyce et al. 2013; Kilduff et al. 2013; but also see, for example, Voracek and Stieger 2009 for a contrary viewpoint). We accordingly speculated that higher prenatal androgen loads increase the risk for a completed suicide later in life. To investigate this hypothesis, we used the second-to-fourth-finger length ratio (2D:4D). 2D:4D is determined by the intrauterine sex hormone milieu during digit cartilage development. It is negatively correlated with prenatal exposure to androgens and is, accordingly, a proxy for measuring prenatal androgen load during adulthood (Breedlove 2010; Zheng and Cohn 2011; Manning et al. 2014). In addition, the difference between the right 2D:4D (R2D:4D) and the left 2D:4D (L2D:4D) (=Dr-l) is suggested to correlate negatively with the prenatal androgen load (for review Manning et al. 2014). Our primary hypothesis was that suicide corpses would exhibit a lower 2D:4D than non-suicide corpses and that the difference would be more pronounced in males than in females. We additionally hypothesized a difference in 2D:4D between suicide corpses of individuals who had died of more-aggressive physical methods and suicide corpses of individuals who had died of less-aggressive chemical methods (poisoning).

## Methods

This study was part of the Finger Length in Psychiatry (FLIP) project of the Erlangen Department of Psychiatry and Psychotherapy (Kornhuber et al. 2011, 2013). Between January 2012 and September 2014, we enrolled 71 corpses (estimated post-mortem interval until enrollment: 7 h–14 days) that were transferred to the Institute for Legal Medicine at the Friedrich-Alexander University Erlangen-Nürnberg for post-mortem examination. An experienced coroner (DT) scanned (Avision IS1000 flatbed scanner, Hsinchu, Taiwan) or photographed (in-house-constructed apparatus with a fixed geometry between the fingers and the camera) the corpses’ right and left hands. The photography method had an advantage over the scanning method under certain circumstances (e.g., distinct rigor mortis), because it was easier to handle. The total lengths of the second (2D) and fourth (4D) digits were quantified from the middle of the basal crease to the finger tips (in pixels using the GNU Image Manipulation Program; GIMP, version 2.8; http://www.gimp.org). Each finger was measured six times by three independent raters who were blind to the group allocation. The mean values were calculated for the R2D:4D, the L2D:4D, and the Dr-l. The one-way random intra-class correlation coefficients (ICC) indicated good interrater reliability (>86 % of the variance in the raters’ means was real; R2D:4D: .88, L2D:4D: .87, Dr-l: .87). Moreover, we found high reliabilities between the scanning method and the photography method (two-way random ICC (absolute agreement) in the data of 20 hands measured with the two methods by three independent raters: R2D:4D: .94, L2D:4D: .93, Dr-l: .77).

We present the data as means and standard errors of the mean (±SEM) or as medians and interquartile range (IQR) if the data did not follow a normal distribution according to the Kolmogorov–Smirnov one-sample tests for goodness of fit. We calculated repeated-measures analyses of variance (ANOVA) with repeated measurements on the factor “hand” (right, left) and separate ANOVAs with R2D:4D, L2D:4D, and Dr-l as dependent variables. Status of alcohol dependence and weight status were included in the statistical models, because these factors are associated with both 2D:4D (Fink et al. 2006; Han et al. 2016; Kornhuber et al. 2011; Oyeyemi et al. 2014) and suicide rate (Perera et al. 2016; WHO 2014). The variables of interest (“group,” suicide versus non-suicide; “method of suicide,” chemical versus physical) and the potential confounders (“quantification method,” scan versus photograph; “status of alcohol dependence,” alcohol-dependent versus not alcohol-dependent versus unknown; “weight status,” normal weight ≤25 mg/m^2^ versus overweight >25 kg/m^2^) were included in the models as fixed factors. Neither age nor the post-mortem interval was significantly correlated with R2D:4D, L2D:4D, or Dr-l (Spearman) in the entire group or in any subgroup (males, females, suicide corpses, non-suicide corpses) and, therefore, were not included into the primary models. The effect size was estimated by partial *η* ^2^ values and is shown for significant associations. *p* values less than .05 for two-sided tests were considered to be statistically significant. We analyzed the data using IBM SPSS statistics Version 21 for Windows (SPSS Inc., Chicago, IL, USA) and Graph Pad Prism 5 (Graph Pad Software Inc., San Diego, CA, USA).

## Results

### Sample description

We characterized the groups of suicide corpses and non-suicide corpses as follows. Suicide corpses (*n* = 46): 32 males, 14 females; 21 overweight, 25 normal weight; 7 alcohol-dependent, 25 not alcohol-dependent, and 14 unknown status of alcohol dependence; 40 scans and 6 photographs; median age 48 years (IQR:32–61 years); median estimated post-mortem interval 48 h (IQR:24–96 h); methods of suicide: chemical-related suicides: 15 self-poisoning, including carbon monoxide; physical-related suicides: 9 hanging/suffocation, 6 self-inflicted shooting death, 6 self-injury cutting, 5 jumping from height, 2 vehicular impact, 2 electrocution, and 1 hypothermia. Non-suicide corpses (*n* = 25): 15 males and 10 females; 13 overweight and 12 normal weight; 3 alcohol dependent and 14 not alcohol dependent, 8 unknown status of alcohol dependence; 20 scans and 5 photographs; median age: 55 years (IQR:53–73 years); median estimated post-mortem interval 84 h (IQR:47–120 h); causes of death: cardiac failure, cerebral hemorrhage, cerebral tumor, traumatic brain injury, accident, hypothermia, intoxication, pulmonary embolism, pneumonia, sepsis, and others.

### 2D:4D and suicide

**Fig. 1:**
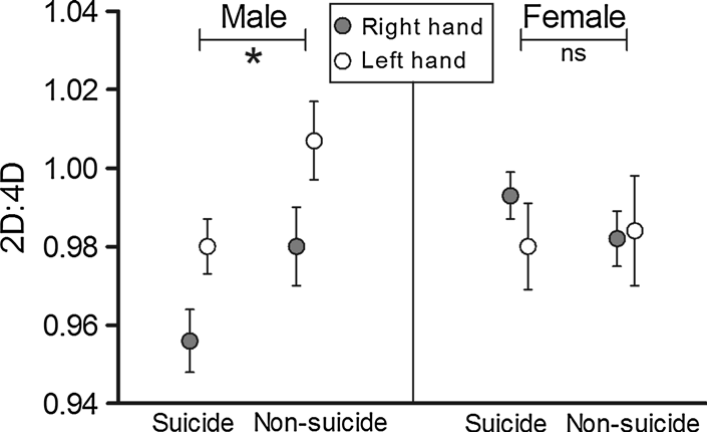
Right- and left-hand second-to-fourth-finger length ratios in male and female suicide corpses and non-suicide corpses. Second-to-fourth-finger length ratios (2D:4D) are significantly lower in suicide corpses than in non-suicide corpses for males (repeated-measures ANOVA, *F* = 5.2, *p* = .030, and partial *η* ^2^ = .147) but not for females (repeated-measures ANOVA, *F* = .3, and *p* = .614). The *graph* shows mean ± SEM. *p < .05; *ns* not significant

Consistent with our primary hypothesis, we found lower 2D:4D in the suicide corpses than in the non-suicide corpses (repeated-measures ANOVA, *F* = 5.1, *p* = .029, partial *η* ^2^ = .111). This effect remained significant after including age and the estimated post-mortem interval as covariates (repeated-measures analysis of covariance (ANCOVA), suicide versus non-suicide, *F* = 5.0, *p* = .031, and partial *η* ^2^ = .114). The two statistical models were also influenced by the hand × sex (repeated-measures ANOVA, *F* = 8.4, *p* = .006, and partial *η* ^2^ = .171; repeated-measures ANCOVA, *F* = 8.8, *p* = .005, and partial η^2^ = .184) and the hand × sex × weight status × status of alcohol dependence interactions (repeated-measures ANOVA, *F* = 5.3, *p* = .027, and partial *η* ^2^ = .114; repeated-measures ANCOVA, *F* = 4.7, *p* = .037, and partial η^*2*^ = .107). There were no additional significant main or interaction effects of 2D:4D with the quantification method (scan versus photograph), the status of alcohol dependence, or weight status. Separate sex-specific repeated-measures ANOVAs revealed that the association between 2D:4D and group (suicide versus non-suicide) was primarily driven by the male subsample (Fig. 1).

Additional exploratory analyses showed that R2D:4D was lower in suicide corpses (.973 ± .005) than in non-suicide corpses (.981 ± .007, ANOVA, *F* = 8.1, *p* = .007, and partial *η* ^2^ = .165) and lower in males (.967 ± .006) than in females (.988 ± .007, *F* = 6.3, *p* = .016, and partial *η* ^2^ = .133). By contrast, L2D:4D did not significantly differ between suicide corpses (.980 ± .006) and non-suicide corpses (.997 ± .008, *F* = 1.6, and *p* = .213). Finally, Dr-l was not significantly different between suicide corpses (−.008 ± .005) and non-suicide corpses (−.017 ± .007, ANOVA, *F* = 2.0, and *p* = .167), but Dr-l was associated with sex (males −.026 ± .006, versus females .006 ± .007, *F* = 8.4, *p* = .006, and partial *η* ^2^ = .171).

### 2D:4D and suicide method

2D:4D did not significantly differ between corpses of individuals who had died from various physical methods (*n* = 31, .972 ± .007) and corpses of individuals who had died from chemical methods (=poisoning, *n* = 15, .979 ± .009, repeated-measures ANOVA, *F* = .4, and *p* = .509). Furthermore, there was no significant association between Dr-l and suicide method (physical methods, −.016 ± .007, poisoning, .002 ± .008; ANCOVA, *F* = 1.6, and *p* = .218).

## Discussion

To the best of our knowledge, this is the first study to investigate 2D:4D, a proxy for prenatal androgen exposure, in death by suicide. We have reported lower 2D:4D ratios in suicide corpses than in non-suicide corpses with partial η^2^ values between .111 and .184. These effect sizes are considered to be medium (>.059) to large (>.138; Richardson 2011). Because 2D:4D correlates negatively with the extent of prenatal exposure to androgens, our results may indicate that higher prenatal androgen load increases the risk for death by suicide. Our findings agree with the assumption of Manning et al. (2014) that prenatal androgen priming exerts its effects in adulthood predominantly in aggressive situations which applies to situations prior to a completed suicide.

Our secondary analyses revealed that the 2D:4D difference between suicide corpses and non-suicide corpses was mainly driven by males. This finding is consistent with other studies that demonstrated a 2D:4D effect in males but not in females for attention deficit hyperactivity disorder symptoms (Martel et al. 2008), fear reactivity (Bergman et al. 2010), and sex-typical behavior (Mitsui et al. 2016). In accordance with Martel et al. (2008), we speculate that sex differences in intrauterine brain development (e.g., biochemical or anatomical aspects) might render males more vulnerable to the effects of prenatal androgenization than females. In addition, if there is a threshold of prenatal androgen load for increased suicide risk, this threshold should be exceeded more often by males than by females because of the sex-specific higher baseline intrauterine androgen load in males. Our secondary analyses also revealed a stronger association between 2D:4D and suicide on the right hand than on the left hand which is in line with findings suggesting that the R2D:4D is superior to the L2D:4D in indicating prenatal androgenization (Hönekopp and Watson 2010; Manning et al. 1998, 2014). In line with our expectations, we further found sexual dimorphisms in R2D:4D and Dr-l with lower values in males than in females

This study has several limitations. It was sufficiently powered to verify the primary hypothesis; however, we were unable to demonstrate an association between 2D:4D and the suicide method, which might be due to us investigating an insufficient number of suicide corpses. Moreover, our study was limited by the fact that the cohort might not be typical of the total cohort of suicides, because we enrolled a random but not necessarily representative subsample of corpses that were transferred to the Institute for Legal Medicine at the Friedrich-Alexander University Erlangen-Nürnberg. Future investigations are needed to test the generalizability of our results. In addition, our results should be interpreted cautiously. Underlying causalities cannot be drawn from the associational study design. Moreover, the accuracy of 2D:4D as a proxy of prenatal androgen exposure may be limited because of known genetic determinants of 2D:4D (Forstmeier 2005; Medland and Loehlin 2008) and other possible influencing and mediating factors. A strength of this investigation was that the statistical models were corrected for potential confounding variables, such as sex, body weight (Oyeyemi et al. 2014), and alcohol dependence (Kornhuber et al. 2011). However, we acknowledge that the inclusion of these confounders entailed small subgroup sample sizes and that we were unable to classify the alcohol dependence status in nearly one-third of the cases. Finally, we did not investigate a potential bias caused by other mental illnesses.

So far, established risk factors cannot reliably determine individuals who will prospectively complete suicide. We expect that our results will improve efforts to both predict and prevent suicides in the future. In combination with other clinical markers and biomarkers, 2D:4D may be useful for effectively predicting suicides and the transition from suicidal ideation to a completed suicide in clinical and non-clinical samples. Because suicide attempts are more often fatal in males than in females and because our study revealed an association between 2D:4D and completed suicide only in males, future studies should examine whether prenatal androgen exposure entails death primarily by facilitating the transition from a suicidal attempt to suicide completion. Moreover, additional studies are necessary to reveal the pathways that link the intrauterine androgen priming and suicide. It might be worthwhile investigating the role of cerebral epigenetic alterations and aggressive and impulsive behavioral phenotypes.

In summary, our findings suggest that higher prenatal exposure to androgens may predispose a male individual to commit suicide later on in his life. This knowledge may aid in establishing novel strategies to predict and prevent suicides in the future.
